# Cross-sectional dataset of low- and semi-skilled gig workers in India: COVID-19 and human security

**DOI:** 10.1016/j.dib.2026.112892

**Published:** 2026-05-29

**Authors:** Bita Afsharinia, Anjula Gurtoo

**Affiliations:** Department of Management Studies, Indian Institute of Science, Bangalore 560012, India

**Keywords:** Gig workers, Human security framework, India, Sociodemographic factors, Fear and apprehensions

## Abstract

The paper presents a cross-sectional dataset of low- and semi-skilled gig workers in India, collected during a single survey wave in the pandemic. Respondents retrospectively reported their experiences at two time points pre-pandemic (July–November 2019) and during the pandemic (December 2020–January 2021) across multiple human security dimensions, framed within the United Nations Human Security Framework (2016). The dataset represents original primary data and captures the economic, food, health, environmental, personal, community, and political experiences of gig workers during the crisis. The dataset focuses specifically on low- and semi-skilled adult gig workers, including drivers, domestic workers, delivery personnel, beauticians, street vendors, small business owners, and self-employed service providers. The dataset has two parts. Part A captures sociodemographic details, employment status, income, loans, COVID-19 impacts on livelihood, food security, health access, living conditions, and government/community support. Part B records fear and apprehensions, including financial security, social support, community tensions, housing issues, and vaccine attitudes. Data were collected using a structured questionnaire and variables independently developed by the authors; community volunteers from SJS (Mitr Sanketa initiative) only facilitated survey administration. The survey covers 136 variables aligned with the UN Human Security Framework (2016), including economic, food, health, environmental, personal, community, and political security. The dataset provides valuable insights for research and education in understanding the vulnerabilities, resilience, and lived experiences of gig workers during crises.

Specifications Table

**Table:** 

Subject	Social Sciences
Specific subject area	Gig economy, COVID-19 impacts, human security, labour conditions, and vulnerabilities of low- and semi-skilled gig workers in urban India.
Type of data	The dataset is provided in tabular form and is available in Excel (XLSX), SPSS (.sav), and a non-proprietary comma-separated values (CSV) format. The dataset includes raw, analysed, and processed data, providing a comprehensive overview of low- and semi-skilled gig workers ’sociodemographic, economic, and COVID-19 related information.
Data collection	The dataset was generated through structured, face-to-face surveys using a questionnaire and variables designed independently by the authors, based on the United Nations Human Security Framework (2016). Data collection was conducted in a single wave during the pandemic using this single structured questionnaire. Respondents were asked to retrospectively recall their experiences at two time points: pre-pandemic (July–November 2019) and during the pandemic (December 2020–January 2021), allowing the study to capture changes over time within one survey. Volunteers from the community-based Trade Union Stree Jagruti Samiti (SJS), operating under the Mitr Sanketa initiative, were involved solely in administering the survey and facilitating access to respondents, without any involvement in the design, ownership, analysis, or interpretation of the data. Inclusion criteria included adults engaged in low- and semi-skilled gig work in the informal sector (low-skilled: domestic workers and street vendors; semi-skilled: drivers, delivery personnel, beauticians, and self-employed service providers). Informed consent was obtained from all participants through the third-party organization (Mitr Sanketa/SJS) as the implementing body responsible for participant outreach and ethical compliance. Data were checked for completeness and consistency before being entered into Excel, SPSS and R for analysis.
Data source location	The primary dataset, collected in Bangalore, India, a metropolitan city in southern India with diverse populations and economic activities was independently generated by the authors using a questionnaire based on the UN Human Security Framework. Data collection was facilitated by the community-based organization Stree Jagruti Samiti under the Mitr Sanketa initiative, which administered the survey without involvement in questionnaire design, data ownership, or analysis.
Data accessibility	All raw data referred to in this article are publicly available in a data repository prior to publication [1,2]. Repository name: Mendeley Data Data identification number: Dataset: 10.17632/j6hd2dfsh8.1; Tables: 10.17632/hyd7mvkrpz.1 Direct URL to data: • Dataset: https://data.mendeley.com/datasets/j6hd2dfsh8/1 • Dataset with original missing values: 10.17632/vms9g9mrfb.1, and https://data.mendeley.com/datasets/vms9g9mrfb/1 • Tables: https://data.mendeley.com/datasets/hyd7mvkrpz/1 Instructions for accessing these data: The dataset and tables are publicly available and can be accessed without login or special permissions using the provided links.
Related research article	Afsharinia, B., & Gurtoo, A. (2024). COVID-19 impact on food consumption of low-skilled employees in India. Global Food Security, 42(1). https://doi.org/10.1016/j.gfs.2024.100791

## 1 Value of the Data

• The survey design is based on the UN Human Security Framework (2016), covering economic, food, health, environmental, personal, community, and political dimensions of human well-being.
• The dataset captures gig workers’ experiences in India at two time points pre-pandemic (July–November 2019) and during the pandemic (December 2020–January 2021) based on retrospective reporting within a single survey, allowing analysis of changes over time and offering a unique perspective on temporal dynamics.
• These data offer a valuable resource for researchers investigating how crises affect multiple aspects of human security, particularly in informal work sectors.
• The dataset enables exploration of the relationships between socio-demographic factors, fears, and vulnerabilities among low- and semi-skilled gig workers.
• Insights from the dataset can support policymakers in designing inclusive, crisis-responsive interventions to safeguard vulnerable populations in future emergencies.
• By capturing retrospective experiences in a single survey wave, the dataset provides a practical and efficient way to compare conditions before and during the pandemic, supporting descriptive and comparative analyses of temporal changes.

## 2 Background

The dataset was compiled to capture the multidimensional effects of crises on gig workers in India, using the United Nations Human Security Framework (2016) as the theoretical foundation. The framework’s economic, food, health, environmental, personal, community, and political dimensions guided the survey design. Data collection was conducted in a single wave during the pandemic (December 2020–February 2021) using a structured questionnaire. Respondents were asked to retrospectively recall their experiences at two time points: pre-pandemic (July–November 2019) and during the pandemic (December 2020–January 2021). The entire data collection process spanned approximately 8 weeks (about 3 months), including recruitment, survey administration, and quality checks. This single-wave approach allowed capturing changes over time without recontacting participants, while minimizing attrition-related concerns.

Structured, face-to-face surveys were conducted with gig workers, including drivers, delivery personnel, domestic workers, beauticians, street vendors, small entrepreneurs, and other self-employed workers. The survey was designed based on the UN Framework to capture multidimensional aspects of household well-being before and during the COVID-19 pandemic. A structured questionnaire was developed independently by the authors to ensure comprehensive coverage of socio-demographic, employment, health, and food security variables. The dataset includes variables related to income, health access, living conditions, social support, fears, and coping strategies. The article complements the published research by providing a detailed description of the dataset’s structure, collection methods, and potential applications, allowing other researchers and policymakers to further explore the dataset for cross-contextual studies, crisis interventions, and socio-economic analyses.

The data were collected directly from low-income gig workers in India, specifically distinguishing between low-skilled workers, who perform routine, manual, or unskilled tasks with minimal formal training, and semi-skilled workers, who perform jobs requiring some technical knowledge, short-term training, or experience without full professional qualifications. The dataset provides a rare comparative insight into the lived experiences of vulnerable urban populations during crises.

To capture the diversity of low- and semi-skilled gig workers, the study employed a mixed sampling strategy combining systematic random sampling and snowball sampling. Systematic random sampling ensured methodological rigor by providing structured coverage of workers accessible through existing records, while snowball sampling enabled inclusion of workers who are often absent from formal lists due to informal or irregular employment arrangements. Integrating both approaches allowed the dataset to reflect the heterogeneity of gig work experiences while maintaining scientific robustness [3].

## 3 Data Description

**Fig. 1:**
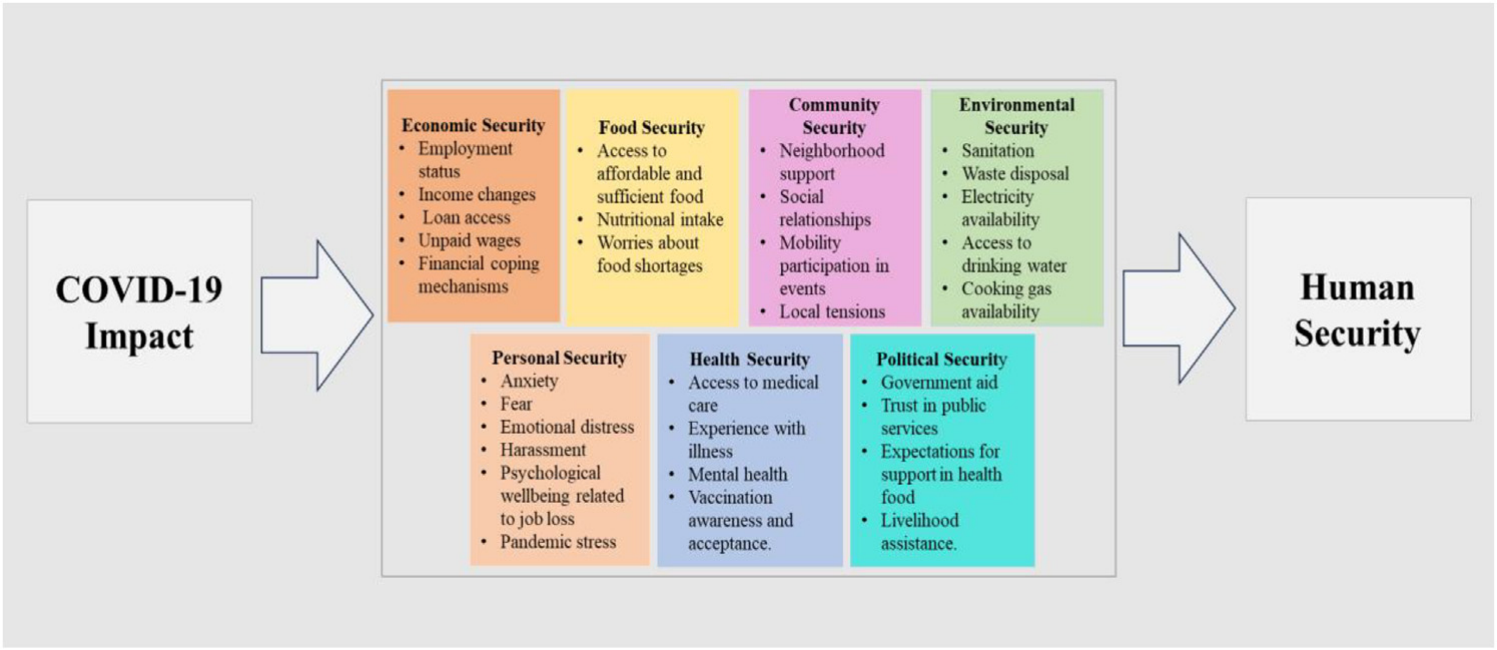
Study variables organized by UN human security dimensions.

The COVID-19 pandemic significantly affected employment and livelihoods across the globe [4]. Gig economy workers, already engaged in precarious and informal employment, faced increased vulnerabilities, including job loss, reduced working hours, and diminished income security [5,6]. Through a single-time survey conducted during the pandemic, in which respondents retrospectively reported on both periods, the COVID-19 and Human Security dataset captures the experiences of low- and semi-skilled gig workers at two reference periods pre-pandemic (July–November 2019) and during the pandemic (December 2020–January 2021). The dataset is structured according to the United Nations Human Security Framework, enabling analysis of multidimensional vulnerabilities and temporal changes among urban gig workers in India. Fig. 1 illustrates the study variables grouped into the UN Human Security dimensions, covering economic, food, health, environmental, personal, community, and political security aspects relevant to vulnerable urban households during the pandemic.

The dataset includes detailed information on gig workers’ sociodemographic characteristics, employment status, income, loans, food and health access, living conditions, government and community support, as well as psychological factors such as fears, apprehensions, and vaccine attitudes. The target respondents comprised low- and semi-skilled gig workers based in Bangalore, India, including low-skilled workers (e.g., domestic workers and street vendors) and semi-skilled workers (e.g., drivers, delivery personnel, beauticians, small entrepreneurs, and other self-employed individuals).

To maintain clarity and readability, the manuscript includes only tables summarizing the basic demographic characteristics and the survey questions with their answer scales. The full questionnaire, along with summary statistics for all 136 variables, is publicly available in the Mendeley Data repository [1], allowing interested researchers to access the complete dataset. The following tables provide descriptive statistics highlighting gig workers’ sociodemographic profiles, economic conditions, and the pandemic’s impact on their livelihoods, household environment, nutrition, and well-being. These tables serve as a structured overview of the dataset.

Table 1 presents descriptive statistics of gig workers’ sociodemographic characteristics, including gender and education level (categorical variables). The minimum and maximum columns reflect the coding range for each categorical variable.

**Table 1:** Descriptive statistics of gig workers’ sociodemographic characteristics.

PART A – About You		Frequency	Percent	Coding range (minimum)	Coding range (maximum)
Gender	Male	1259	44.5%	1	2
	Female	1571	55.5%	1	2
	Total	2830	100.0		
Education	No education	619	21.9%	0	5
	Primary (grades 1 to 5)	317	11.2%	0	5
	Middle school (grades 6 to 8)	450	15.9%	0	5
	Secondary (grades 9 and 10)	631	22.3%	0	5
	Highschool/PUC (grades 11 and 12)	488	17.2%	0	5
	College above (undergraduate, diplomas, post graduate, masters and PhD)	325	11.5%	0	5
	Total	2830	100.0		

Note: Minimum and maximum values indicate the possible coding range of each variable and not the observed minimum and maximum values in the sample.

Next, Table 2 presents descriptive statistics of sociodemographic and family economic characteristics for numerical variables. Respondents had an average age of 37 years old (range: 18–80). Households consisted of about 4–5 members (M = 4.65, SD = 1.67), with nearly two members employed before COVID-19 (M = 1.93, SD = 1.09), with declined to fewer during the pandemic (M = 1.68, SD = 0/99). The average monthly household income in 2021 stood at ₹18,903 (SD = 14,474; range: 0–300,000).

**Table 2:** Descriptive statistics of sociodemographic and family economic characteristics.

		Mean						
Variables	N Statistic	Statistic	Std. Error	Std. Deviation	Range Statistic	Min Statistic	Max Statistic	Sum Statistic
Age	2830	36.86	0.18	9.684	62	18	80	104,309
Total family members	2830	4.65	0.03	1.669	14	1	15	13,163
Family member employed before COVID	2830	1.93	0.02	1.087	9	0	9	5456
Family members employed now	2830	1.68	0.02	0.99	6	0	6	4754
FAMILY monthly earning now-2021	2830	18,903.1	272.08	14,474.28	300,000	0	300,000	53,495,765

Table 3 summarizes the survey questions in the dataset, providing a clear overview of the variables and response scales used for data collection. Detailed summary statistics and additional analyses are provided in the supplementary materials. The full dataset and further statistical information are available in Mendeley.

**Table 3:** Survey questions and response scales.

Domain	Variable / Question	Response scale
**Economic Characteristics**	Currently employed	Yes / No
	Current job type	Driver / Delivery / Domestic work / Street vending / Beauty services / Small business / Self-employed / Not working
	Job before COVID-19	Same categories as above
	Reason for unemployment / no earnings	COVID-19 / Lockdown / No work / Health issues / Not applicable
	Paid for work done before lockdown	Yes / No
	Mortgaged vehicle/gold	Yes / No
	Took loan	Yes / No
	Reason for loan	Food / Health / Education / Rent / No work / Vehicle / Family reasons / Not applicable
	Source of loan	Friends/relatives / Bank/finance / Private lender / Not mentioned
	Loan amount	≤₹10,000 / ₹11,000–20,000 / ₹21,000–30,000 / >₹30,000
	Interest rate	≤5% / 6–10% / 11–15% / 16–20% / >20% / Not applicable
**Income & Housing**	Monthly earnings	Continuous (₹)
	Persons per room	Continuous (count)
**Livelihood Impact**	Children’s school fees paid	Yes / No
	Access to online classes	Yes / No
	Child dropout	Yes / No
	Eating less food	Yes / No
	Meals reduced	Yes / No
	Source of food	Ration shop / Self-purchase / Private donation / Government donation
	Bought clothes (self/family)	Yes / No
	Doctor visit	Yes / No
	Type of healthcare	Private / Government / None
	COVID infection in family	Yes / No
	Received medical/community/government support	Yes / No
	Paid rent	Yes / No
	Changed house/locality	Yes / No
	Got old job back	Yes / No
	Learned new skill/technique/technology	Yes / No
**Environment**	Garbage collection	Yes / No
	Electricity access	Yes / No
	Bathroom access	Yes / No
	Water access	Yes / No
	Cooking gas availability	Yes / No
**Food & Nutrition**	Family slept hungry	Yes / No
	Food affordability	Specific food groups / Multiple groups / None
	Worry about food availability	Financial / Health fear / Future worry / No
	Food frequency (rice, milk, dal, veg, non-veg)	Daily / 1–3 times per week / Weekly
**Personal & Community**	Increased use of technology at work	Yes / No
	Mobile use	News / TV / Videos
	Job changes (wages, workload, deposits)	Yes / No
	Emotional wellbeing	No / A little / Yes
	Government support	Ration / Medical / Cash / Helpline / None
	Expected government support	Food / Medical / Financial / Nothing
**Fears & Apprehensions**	Wage increase expectation	5-point Likert (No–Yes)
	Loan repayment ability	5-point Likert
	Job security fear	5-point Likert
	Neighbour/community support	Yes / No (emotional, food, money, care)
	Community tensions, violence, drinking	5-point Likert
	Technology anxiety	5-point Likert
	Mobility & social participation	5-point Likert
	Landlord harassment	5-point Likert
**Vaccine Attitudes**	Vaccine awareness	Yes / No
	Belief in vaccine effectiveness	Yes / No / Don’t know
	Knowledge of vaccination site	Yes / No
	Willingness to vaccinate	Yes / No

## 4 Experimental Design, Materials and Methods

The dataset is organized into five main sections: (A) study setting and population, (B) study design and sampling, (C) data collection process, (D) survey coding, and (E) data processing and analysis.

(A) Study Setting and Population

The research was conducted in Bangalore, India, a metropolitan city in southern India with considerable socioeconomic diversity [7]. The target population comprised low- and semi-skilled gig workers, such as cab drivers, domestic workers, delivery personnel, beauticians, street vendors, small business owners, and self-employed workers including masons, plumbers, and carpenters.

Low-skilled gig workers are defined as individuals engaged in routine, predominantly manual or service-based tasks that require little to no formal education or certified training [5,8]. Semi-skilled gig workers refer to individuals employed in occupations that involve basic technical competencies, task-specific skills, or experiential learning acquired through short-term training or on-the-job experience, but that do not require formal professional qualifications or advanced credentials [9,10]. These groups were identified as vulnerable due to insecure employment and limited access to social protections during the COVID-19 pandemic [5,11].

(B) Study Design and Sampling

A cross-sectional design with retrospective recall was adopted to capture experiences at two time points: pre-pandemic (July–November 2019) and during the pandemic (December 2020–January 2021). Data collection was conducted in a single wave using the same structured questionnaire, allowing respondents to retrospectively report their experiences for both periods and ensuring consistency in measurement.

Although the study spans two temporal periods, the study did not adopt a panel design; participants were not recontacted, and all responses were collected in a single survey. During the pandemic phase, respondents reported their current situation and retrospectively recalled their pre-pandemic conditions using identical items. COVID-specific questions covering pandemic-related disruptions such as lockdown impacts, COVID-related job loss, health fears, and access constraints were administered only for the pandemic phase. This design enables comparison of perceived changes over time while avoiding panel attrition, though the design relies on retrospective self-report rather than repeated observation of the same individuals.

For the purposes of the study, gig workers are defined as individuals engaged in task-based, platform-mediated, or informal service work characterized by income insecurity, limited contractual protection, and absence of formal social security [12,13]. Low-income gig workers refer to individuals whose primary source of livelihood is derived from such work and who operate within the lower income strata of the urban informal economy [14]. Low-skilled gig workers include occupations requiring minimal formal training or certification (e.g., domestic workers and street vendors), while semi-skilled gig workers include occupations requiring basic technical skills or experience but limited formal qualifications (e.g., drivers, beauticians, small entrepreneurs, and other self-employed service providers) [15]. These definitions are applied consistently throughout the manuscript.

To balance accessibility and methodological rigor, the study employed a combination of systematic random sampling and snowball sampling. For systematic random sampling, the frame consisted of worker lists maintained by the community-based organization Stree Jagruti Samiti (SJS) under the Mitr Sanketa initiative. These lists covered low-income, informal-sector workers in Bangalore engaged in occupations such as driving, delivery services, domestic work, street vending, beauty services, and small self-owned businesses. Eligible individuals were then selected at fixed intervals following a random start, ensuring unbiased representation [3].

Snowball sampling was used as a complementary strategy to reach hard-to-access workers not fully captured in organizational records, including individuals with irregular or platform-based employment (e.g., app-based drivers, delivery workers, beauty service providers), high mobility (e.g., frequent changes in residence or worksites), unstable income (e.g., irregular daily wages or large month-to-month income fluctuations), or livelihoods disrupted during the COVID-19 period due to job loss, temporary work suspension, or occupational shifts [4]. The integration of probability-based systematic random sampling with snowball sampling is methodologically appropriate in contexts where the target population is identifiable but access is constrained, and has been widely applied in studies of informal and hard-to-reach populations [5,6].

The final dataset comprised 2830 respondents aged between 18 and 80 years (mean age = 37, SD = 10). A total of 2837 questionnaires were administered, of which seven were excluded due to incompleteness, resulting in an effective participation rate of approximately 99.8% (calculated as 2830 ÷ 2837 × 100). The extent of missing values for each variable is reported in Appendix 1. Inclusion criteria required employment status (91% employed in 2020–21; 9% unemployed) and engagement in the gig economy (low- and semi-skilled). The sample included 44.5% male and 55.5% female respondents. Because participant recruitment involved a mixed sampling strategy, including snowball referrals, the total number of individuals initially approached but declining participation could not be fully enumerated; therefore, representativeness cannot be statistically inferred. Nevertheless, the final sample spans multiple gig-economy occupations and income strata among urban gig workers in Bangalore, providing broad descriptive coverage of the study population.

(C) Data Collection Process

Primary data were collected using a structured survey questionnaire consisting exclusively of closed-ended items with fixed response options (binary, categorical, and Likert-scale). Data were collected using a structured questionnaire developed by the authors and administered face-to-face to facilitate clarity, comprehension, and consistency of responses. The questionnaire and all study variables were independently developed by the authors based on the United Nations Human Security Framework (2016) [16]. Community-based organizations, Stree Jagruti Samiti (SJS), under the Mitr Sanketa initiative, were engaged solely to administer the survey and facilitate respondent access; they had no role in questionnaire design, data ownership, analysis, or interpretation.

The survey was administered in person by trained volunteers from SJS, who read questions verbatim from the questionnaire and wrote down respondents’ answers. All responses were recorded manually on paper-based questionnaires at the time of survey administration. No audio, video, or electronic recordings were used. Each administration lasted approximately 20 min. Volunteers, fluent in local languages and familiar with community contexts, facilitated communication and trust with respondents.

Informed consent was obtained from all participants prior to participation. As the implementing organization responsible for participant outreach and ethical compliance during data collection, Mitr Sanketa/SJS administered the informed consent process. The consent statement was often read aloud to participants in the local languages (Kannada and Hindi) to ensure comprehension. The consent text was as follows:

### 4.1 Consent to participate

“You are invited to take part in this survey for academic research purposes (academic publications, reports, or presentations). Participation is voluntary. We confirm that your responses are confidential and researchers will not be able to link them to your identity in any form. By proceeding, you confirm that you have understood this information and voluntarily consent to participate.”

☐ I agree to participate

The ‘consent to participate’ was often read out loud to the participant in the local language of Kannada and Hindi. Ethical approval for field implementation was obtained through Mitr Sanketa, and the approval letter included in the Supplementary Materials confirms the organization’s authorization for the authors to use the collected data exclusively for academic research purposes. The authors retain full ownership of the dataset and full responsibility for the study design, data analysis, interpretation, and reporting.

The questionnaire was originally developed in English and translated into Kannada and Hindi. Survey administration was conducted in the respondent’s preferred language to minimize language-related response bias and ensure comprehension. First, the questionnaire included fully written-out questions (e.g., “Did you buy clothes last year?”) and brief prompts (e.g., “Community support if got COVID?”) to focus on key variables while allowing clarifications during face-to-face administration. Although it did not contain full transition scripts, all volunteers from Stree Jagruti Samiti (SJS) were extensively trained to follow standardized procedures, clarify prompts consistently, and maintain neutral phrasing. Importantly, interviewers were not free to rephrase questions arbitrarily.

All survey administrators were volunteers from Stree Jagruti Samiti (SJS) and underwent extensive standardized training prior to data collection. This training consisted of:

I. Two dedicated training sessions (2 + 2 h) jointly conducted by the research team and SJS,
II. Detailed explanations of the study objectives, context, and importance of each questionnaire section,
III. Clear instructions on how each prompt should be verbally expanded into a complete, standardized question (e.g., “Did you receive any community support if you got COVID-19?”),
IV. Emphasis on maintaining neutral phrasing and avoiding any form of respondent priming.

In addition, mock interviews and demonstrations were conducted during training, where volunteers practiced administering the questionnaire by first interviewing the research team and then repeating the same questions verbatim. During the initial phase of data collection, members of the research team were present on-site to monitor interviews and ensure adherence to standardized procedures. Field supervisors further oversaw data collection to minimize interviewer variability.

Although the questionnaire did not include explicit written transition scripts between sections, transitions were also covered during training to ensure uniform movement between sections without leading respondents. Second, despite the use of abbreviated prompts in the written instrument due to spacing limitations, the combination of structured training, supervision, and monitoring ensured consistent, reliable, and scientifically rigorous data collection across interviewers.

The questionnaire was structured into two parts. Part A collected sociodemographic and economic information, including employment status, income changes before and during the pandemic, loan details, household impacts such as children’s education, food access, healthcare availability, housing, and basic amenities. Part B addressed psychosocial and community aspects, including fears and apprehensions related to wages, job security, loan repayment, social tensions, neighbor support, mobility, technology anxieties, family disruptions, and vaccine perceptions through both categorical and Likert-scale items (1–5).

These data represent original primary data. All variables were independently developed by the authors based on the United Nations Human Security Framework (2016), covering economic, food, health, environmental, personal, community, and political dimensions. The dataset is unique in capturing responses from low- and semi-skilled gig workers including drivers, delivery personnel, domestic workers, street vendors, beauticians, and small business owners through a single survey that asked respondents to retrospectively report experiences at two time points: pre-pandemic (July–November 2019) and during the pandemic (December 2020–January 2021). The dataset provides valuable insights for researchers and policymakers, enabling analysis of multidimensional vulnerabilities, temporal changes, and the lived experiences of populations most affected by the COVID-19 crisis.

(D) Survey Coding

The questionnaire employed a structured coding format to standardize data collection and analysis:

Categorical coding (Y/N): Employment status, access to services (e.g., medical care, education, food), and responses about changes in livelihood or household environment were coded using “1 = Yes” and “0 = No”.

Multiple-choice coding: Responses regarding job types, sources of income, access to government schemes, and vaccination beliefs were coded with predefined answer choices.

Likert-scale coding (1–5): Questions on fears, job security, community tensions, and psychological states were coded using a five-point scale ranging from “Very Easy” to “Very Difficult” or “Yes” to “No” with intermediate options such as “Mostly Yes,” “Not Sure,” and “Mostly Not”.

Frequency coding: Food consumption patterns and medical visits were coded with options such as “Daily,” “2–3 times a week,” “Weekly,” or numeric ranges like “1–2 visits”, “3–4 visits”, and “>4 visits.”

Open numeric coding: Age, income amounts, family size, and duration-related questions were coded as direct numerical entries.

(E) Data Processing

During data cleaning, several types of inconsistencies were addressed. These included out-of-range values (e.g., negative income values or ages reported outside the eligible range of 18–80 years), duplicate entries (identified through matching combinations of survey identifiers and key demographic characteristics), and formatting or coding errors (e.g., age–employment mismatches or obvious data entry errors). The frequency of such issues was low, affecting <1–2% of observations overall, and no single variable exceeded this threshold. Cases with severe issues were excluded prior to analysis, while minor issues were corrected using predefined coding rules (e.g., recoding an age entered as “222″ to “22”). The extent of missing data was systematically assessed using descriptive statistics in SPSS version 25, with the number and percentage of missing observations calculated for each variable (see Appendix 1). This assessment informed the subsequent missing-data handling strategy.

Missing values were addressed using Predictive Mean Matching (PMM) implemented in R. PMM is a semi-parametric imputation method that replaces missing observations with observed values from cases with similar predicted means obtained from regression models. For each variable with missing data, a regression model was fitted using relevant predictors, predicted values were generated for missing observations, and a set of observed cases with the closest predicted values was identified. One observed value from this donor set was then randomly selected to impute the missing value. This approach preserves the original data distribution, variability, and inter-variable relationships, and is particularly suitable for skewed or non-normal data. PMM was implemented using the mice package in R (method = "pmm"), which applies multiple imputation by chained equations.

Both the original dataset with missing values retained (unimputed) and the imputed dataset used for analysis are publicly available, allowing other researchers to apply alternative missing-data strategies if desired.

Frequency analyses were conducted to summarize key variables (see Table 1, Table 2), along with cross-tabulations examining respondents’ sociodemographic characteristics in relation to reported fears, livelihood impacts, and household conditions across the recall periods. Summary statistics were generated using descriptive analytical methods (see Supplementary Materials).

For data management and analysis, Excel, SPSS, and R were used. Excel was employed for initial data entry, formatting, and verification; SPSS was used for descriptive statistics, frequency analyses, and cross-tabulations; and R was used exclusively for missing-data imputation. The final dataset provides a comprehensive account of the economic, social, environmental, health, and psychological impacts of the COVID-19 pandemic on vulnerable gig workers in an urban setting.

## Limitations

The dataset has limitations related to the collection and scope. First, data were collected exclusively in Bangalore, India, which limits generalizability to other urban settings and rural contexts. Second, the use of snowball sampling alongside random sampling introduces selection bias, suggesting future studies could adopt alternative strategies to improve representativeness.

A further limitation is that a conventional response rate could not be precisely calculated because the mixed sampling design particularly snowball recruitment does not permit full enumeration of all individuals initially approached. As a result, the representativeness of the sample cannot be statistically inferred. Nevertheless, the final sample of 2830 respondents spans multiple occupational categories and income strata among low- and semi-skilled gig-economy workers in Bangalore, and the findings should therefore be interpreted as descriptively informative rather than statistically representative.

Third, self-reported responses, particularly regarding income, food access, and fears, are affected by recall or social desirability bias. Furthermore, data were collected in a single wave during the pandemic, and respondents retrospectively reported their experiences before and during the COVID-19 pandemic. This approach allows temporal comparison but may increase recall bias, particularly for pre-pandemic conditions. In addition, although interviewers were trained and followed standardized procedures, the questionnaire included shorthand prompts rather than full scripts, which may have introduced minor variability in phrasing or transitions between sections. Some information is incomplete due to non-responses or missing entries, addressed using predictive mean matching.

Finally, the sample focuses on low- and semi-skilled gig workers; therefore, the experiences of highly skilled or formally employed workers are not represented. Despite these limitations, the dataset provides valuable insights into the multidimensional human security impacts of the COVID-19 pandemic among vulnerable urban gig workers.

## Ethics Statement

The study involved human participants, and informed consent was obtained from all respondents prior to data collection. Mitr Sanketa conducted the survey through their trained volunteers, and the informed consent from participant was obtained by Mitr Sanketa as the implementing organization responsible for participants in local languages when necessary to ensure comprehension. The research was conducted in accordance with the Declaration of Helsinki. Ethical approval for field implementation and data collection was obtained through the Mitr Sanketha initiative, which oversaw the survey administration. The English version of the informed consent form is provided as a Supplementary File. The dataset consist of original primary data, and the third-party organization was involved solely in survey administration, with no role in questionnaire design, data ownership, data analysis, interpretation, or reporting.

## CRediT Author Statement

Bita Afsharinia: Conceptualization, Methodology, Data curation, Formal analysis, Writing original draft, Visualization.

Anjula Gurtoo: Supervision, Conceptualization, Validation, Writing- Reviewing and Editing.

## Data Availability

Mendeley DataCOVID-19 and Human Security (Original data).

## Appendix Supplementary materials

Image, application 1

Image, application 2
