# Supplementary material for: Cross-sectional dataset of low- and semi-skilled gig workers in India: COVID-19 and human security
Source: Data Brief. 2026 May 29;66:112892. doi: 10.1016/j.dib.2026.112892 (PMC13247571; doi:10.1016/j.dib.2026.112892)
Supplement: Supplementary file 1 [file mmc1.pdf]

Date: 22/02/2022

To: Bita Afsharinia

Phone: 919740917137

Email: bitaa@iisc.ac.in

From: Mitr Sanketha

Re: Approval to use Survey – Human Security and COVID-19

Dear Bita Afsharinia,

I hereby to grant permission to Bita Afsharinia, (SR number 05-10-00-91-12-18-2-16515) a Senior Research Scholar (Ph D student) at Indian Institute of Science, Bangalore, India, to use the "Human Security and COVID-19" Survey Datasets for conducting her research entitled, "Study of ecological system determinant of household food security and vulnerability in crisis-prone populations during Covid pandemic in India".

The Institutional Review Board (IRB) of Indian Institute of Science approved the use of the dataset for the purpose of statistical reporting and analysis, and for the registered research. The condition and duration of this approval is for publication purpose only and specified in the policies of Indian Institution of Science, Bangalore, India.

Sincerely,

Name: Geeta Menon

MITR SANKETHA
980/04-05
503, Usha Kiran-25,
Haudin Road, Halasuru
BANGALORE - 560 042

Designation: Secretary, Mitr Sanketa

The Survey Name: Human Security and COVID-19
