# Supplementary material for: Cross-sectional dataset of low- and semi-skilled gig workers in India: COVID-19 and human security
Source: Data Brief. 2026 May 29;66:112892. doi: 10.1016/j.dib.2026.112892 (PMC13247571; doi:10.1016/j.dib.2026.112892)
Supplement: Supplementary file 2 [file mmc2.docx]

*Table 4 shows the economic characteristics of gig workers, covering their employment status, nature of work before and during COVID-19, reasons for unemployment, payment delays, asset mortgaging, and loan-taking behaviour. The table also captures details on sources, purposes, and interest rates of loans, highlighting the financial pressures experienced during the pandemic.*

*Table 4 Descriptive statistics of gig workers’ economic characteristics.*

| *PART A* ***–*** *Your Economics* | | ***Frequency*** | ***Percent*** | ***Minimum*** | ***Maximum*** |
| --- | --- | --- | --- | --- | --- |
| *Currently Employed?* | *No* | *254* | *9.0%* | *0* | *1* |
|  | *Yes* | *2576* | *91.0%* | *0* | *1* |
|  | *Total* | *2830* | *100.0* |  |  |
| *If yes, what is your job?* | *Not work/nothing* | *37* | *1.3%* | *0* | *7* |
|  | *Drivers* | *511* | *18.1%* | *0* | *7* |
|  | *Delivery* | *501* | *17.7%* | *0* | *7* |
|  | *Beauty and other services* | *111* | *3.9%* | *0* | *7* |
|  | *Street vendors* | *719* | *25.4%* | *0* | *7* |
|  | *Domestic workers* | *710* | *25.1%* | *0* | *7* |
|  | *Small Business* | *169* | *6.0%* | *0* | *7* |
|  | *Self-employed* | *72* | *2.5%* | *0* | *7* |
|  | *Total* | *2830* | *100.0* |  |  |
| *Job before COVID?* | *Not work/nothing* | *489* | *17.3%* | *0* | *7* |
|  | *Drivers* | *486* | *17.2%* | *0* | *7* |
|  | *Delivery* | *459* | *16.2%* | *0* | *7* |
|  | *Beauty and other services* | *45* | *1.6%* | *0* | *7* |
|  | *Street vendors* | *378* | *13.4%* | *0* | *7* |
|  | *Domestic workers* | *725* | *25.6%* | *0* | *7* |
|  | *Small Business* | *168* | *5.9%* | *0* | *7* |
|  | *Self-employed* | *80* | *2.8%* | *0* | *7* |
|  | *Total* | *2830* | *100.0* |  |  |
| *If unemployed/no earning 2020-Why?* | *No reason* | *517* | *18.3%* | *0* | *4* |
|  | *Because of covid* | *648* | *22.9%* | *0* | *4* |
|  | *Because of lockdown* | *1098* | *38.8%* | *0* | *4* |
|  | *No job/work* | *557* | *19.7%* | *0* | *4* |
|  | *Because of health issues/accident* | *10* | *0.4%* | *0* | *4* |
|  | *Total* | *2830* | *100.0* |  |  |
| *Did you get paid for work done before lockdown?* | *No* | *1352* | *47.8%* | *0* | *1* |
|  | *Yes* | *1478* | *52.2%* | *0* | *1* |
|  | *Total* | *2830* | *100.0* |  |  |
| *Did you mortgage your vehicle/auto/gold during the lockdown or after the lockdown?* | *No* | *2475* | *87.5%* | *0* | *1* |
|  | *Yes* | *355* | *12.5%* | *0* | *1* |
|  | *Total* | *2830* | *100.0* |  |  |
| *Did you take a loan during March-Aug2020?* | *No* | *1901* | *67.2%* | *0* | *1* |
|  | *Yes* | *929* | *32.8%* | *0* | *1* |
|  | *Total* | *2830* | *100.0* |  |  |
| *If yes, taken loan-why?* | *No* | *2188* | *77.3%* | *0* | *8* |
|  | *Because of food* | *88* | *3.1%* | *0* | *8* |
|  | *Health/medical issues/illness/covid/delivery/ hospital* | *46* | *1.6%* | *0* | *8* |
|  | *Children education/online classes/ fees/ to buy device for online classes* | *130* | *4.6%* | *0* | *8* |
|  | *Because of no work/job/ financial issues/business reasons* | *54* | *1.9%* | *0* | *8* |
|  | *Changing house/building/house rent* | *198* | *7.0%* | *0* | *8* |
|  | *Buy vehicle/auto/bike* | *19* | *0.7%* | *0* | *8* |
|  | *Daily expenses* | *14* | *0.5%* | *0* | *8* |
|  | *Engagement/family problem/family situation* | *93* | *3.3%* | *0* | *8* |
|  | *Total* | *2830* | *100.0* |  |  |
| *From Who taken loan?* | *Not mentioned* | *2101* | *74.2%* | *0* | *3* |
|  | *Friends/relatives* | *359* | *12.7%* | *0* | *3* |
|  | *Bank/finance* | *279* | *9.9%* | *0* | *3* |
|  | *Private* | *91* | *3.2%* | *0* | *3* |
|  | *Total* | *2830* | *100.0* |  |  |
| *How much taken loan?* | *Not mentioned* | *2056* | *72.7%* | *0* | *4* |
|  | *Less than equal to ₹10,000* | *227* | *8.0%* | *0* | *4* |
|  | *₹11,000 to ₹20,000* | *210* | *7.4%* | *0* | *4* |
|  | *₹21,000 to ₹30,000* | *127* | *4.5%* | *0* | *4* |
|  | *Above 30,000* | *210* | *7.4%* | *0* | *4* |
|  | *Total* | *2830* | *100.0* |  |  |
| *Rate of Interest?* | *Not taken loan* | *2129* | *75.2%* | *0* | *5* |
|  | *Less than equal to 5%* | *328* | *11.6%* | *0* | *5* |
|  | *6 to 10%* | *83* | *2.9%* | *0* | *5* |
|  | *11 to 15%* | *36* | *1.3%* | *0* | *5* |
|  | *16 to 20%* | *22* | *0.8%* | *0* | *5* |
|  | *above 20%* | *232* | *8.2%* | *0* | *5* |
|  | *Total* | *2830* | *100.0* |  |  |

*Table 5 reveals the economic characteristics of gig workers based on numerical variables. The table captures changes in monthly earning before COVID-19, during the lockdown, and in late 2020-21, along with household overcrowding measured by the number of people per room during the lockdown.*

*Table 5 Descriptive statistics of economic characteristics at individual level.*

|  |  | | ***Mean*** | | | |  | |  | |  | |  | |  | |
| --- | --- | --- | --- | --- | --- | --- | --- | --- | --- | --- | --- | --- | --- | --- | --- | --- |
| *Variables* | ***N***  ***Statistic*** | | ***Statistic*** | | ***Std. Error*** | | ***Std. Deviation*** | | ***Range***  ***Statistic*** | | ***Min***  ***Statistic*** | | ***Max***  ***Statistic*** | | ***Sum***  ***Statistic*** | |
| *Your monthly earnings now (Dec20_Jan2021)* | | *2830* | | *12023.56* | | *194.99* | | *10373.41* | | *95000* | | *0* | | *95000* | | *34026665* |
| *Monthly earning before COVID?* | | *2830* | | *14604.95* | | *224.64* | | *11950.24* | | *80000* | | *0* | | *80000* | | *41332000* |
| *Monthly earning during lockdown?* | | *2830* | | *3037.92* | | *124.41* | | *6618.54* | | *90000* | | *0* | | *90000* | | *8597300* |
| *How many people stayed in 1 room during lockdown?* | | *2830* | | *3.05* | | *0.034* | | *1.816* | | *11* | | *1* | | *12* | | *8641* |

*Table 6 shows the impact of COVID-19 on households’ livelihood, covering areas such as children’s education, food security, clothing, healthcare access, housing, employment, and skill development. The table highlights how the pandemic affected daily life, access to services, and opportunities for learning and work.*

*Table 6* *Descriptive statistics of the impact of COVID-19 on households’ livelihood*

| *PART A* ***–*** *Impact of Covid on Livelihood* | | ***Frequency*** | ***Percent*** | ***Minimum*** | ***Maximum*** |
| --- | --- | --- | --- | --- | --- |
| *Education of Children-fee paid* | *No* | *1441* | *50.9%* | *0* | *1* |
|  | *Yes* | *1389* | *49.1%* | *0* | *1* |
|  | *Total* | *2830* | *100.0* |  |  |
| *Did children have online classes?* | *No* | *1137* | *40.2%* | *0* | *1* |
|  | *Yes* | *1693* | *59.8%* | *0* | *1* |
|  | *Total* | *2830* | *100.0* |  |  |
| *Were children able attend online classes?* | *No* | *1363* | *48.2%* | *0* | *1* |
|  | *Yes* | *1467* | *51.8%* | *0* | *1* |
|  | *Total* | *2830* | *100.0* |  |  |
| *If not able attend class-why?* | *No money/fee not paid* | *180* | *6.4%* | *1* | *3* |
|  | *No mobile phones* | *20* | *0.7%* | *1* | *3* |
|  | *No children/small kids/other reasons* | *2630* | *92.9%* | *1* | *3* |
|  | *Total* | *2830* | *100.0* |  |  |
| *Did child drop out of school?* | *No* | *2286* | *80.8%* | *0* | *1* |
|  | *Yes* | *544* | *19.2%* | *0* | *1* |
|  | *Total* | *2830* | *100.0* |  |  |
| *Eating less quantity of food?* | *No* | *1446* | *51.1%* | *0* | *1* |
|  | *Yes* | *1384* | *48.9%* | *0* | *1* |
|  | *Total* | *2830* | *100.0* |  |  |
| *No of meals per day SAME or LESS?* | *No* | *2572* | *90.9%* | *0* | *1* |
|  | *Yes* | *258* | *9.1%* | *0* | *1* |
|  | *Total* | *2830* | *100.0* |  |  |
| *Where did you get food from during lockdown-Ration shop by BPL card* | *No* | *1012* | *35.8%* | *0* | *1* |
|  | *Yes* | *1818* | *64.2%* | *0* | *1* |
|  | *Total* | *2830* | *100.0* |  |  |
| *Where did you get food from during lockdown-Bought Myself* | *No* | *2403* | *84.9%* | *0* | *1* |
|  | *Yes* | *427* | *15.1%* | *0* | *1* |
|  | *Total* | *2830* | *100.0* |  |  |
| *Where did you get food from during lockdown-Donation from private people* | *No* | *2025* | *71.6%* | *0* | *1* |
|  | *Yes* | *805* | *28.4%* | *0* | *1* |
|  | *Total* | *2830* | *100.0* |  |  |
| *Where did you get food from during lockdown-Donation from government* | *No* | *2747* | *97.1%* | *0* | *1* |
|  | *Yes* | *83* | *2.9%* | *0* | *1* |
|  | *Total* | *2830* | *100.0* |  |  |
| *Did you buy clothes last year?* | *No* | *1930* | *68.2%* | *0* | *1* |
|  | *Yes* | *900* | *31.8%* | *0* | *1* |
|  | *Total* | *2830* | *100.0* |  |  |
| *Did you buy clothes last year-Children* | *No* | *2369* | *83.7%* | *0* | *1* |
|  | *Yes* | *461* | *16.3%* | *0* | *1* |
|  | *Total* | *2830* | *100.0* |  |  |
| *Did you buy clothes last year-Self* | *No* | *2583* | *91.3%* | *0* | *1* |
|  | *Yes* | *247* | *8.7%* | *0* | *1* |
|  | *Total* | *2830* | *100.0* |  |  |
| *Did you buy clothes last year-Women* | *No* | *2573* | *90.9%* | *0* | *1* |
|  | *Yes* | *256* | *9.0%* | *0* | *1* |
|  | *Total* | *2830* | *100.0* |  |  |
| *Did you buy clothes last year-Old family members* | *No* | *2715* | *95.9%* | *0* | *1* |
|  | *Yes* | *115* | *4.1%* | *0* | *1* |
|  | *Total* | *2830* | *100.0* |  |  |
| *Did you/family visit a doctor during lockdown?* | *No* | *1732* | *61.2%* | *0* | *1* |
|  | *Yes* | *1098* | *38.8%* | *0* | *1* |
|  | *Total* | *2830* | *100.0* |  |  |
| *If yes-Private or Government* | *Non* | *1991* | *70.4%* | *0* | *2* |
|  | *Private* | *567* | *20.0%* | *0* | *2* |
|  | *Government* | *272* | *9.6%* | *0* | *2* |
|  | *Total* | *2830* | *100.0* |  |  |
| *For what problem did you / family member go to the doctor?* | *None* | *1991* | *70.4%* | *0* | *2* |
|  | *COVID symptoms like fever/cough/cold/covid test* | *669* | *23.6%* | *0* | *2* |
|  | *Other health problems* | *170* | *6.0%* | *0* | *2* |
|  | *Total* | *2830* | *100.0* |  |  |
| *No of visits to doctor?* | *None* | *1640* | *58.0%* | *0* | *3* |
|  | *1-2 times* | *848* | *30.0%* | *0* | *3* |
|  | *3-4 times* | *219* | *7.7%* | *0* | *3* |
|  | *More than 4 times* | *123* | *4.3%* | *0* | *3* |
|  | *Total* | *2830* | *100.0* |  |  |
| *Did anyone in your family get COVID?* | *No* | *2636* | *93.1%* | *0* | *1* |
|  | *Yes* | *194* | *6.9%* | *0* | *1* |
|  | *Total* | *2830* | *100.0* |  |  |
| *How was your experience?* | *Not mentioned* | *2181* | *77.1%* | *0* | *3* |
|  | *Bad* | *492* | *17.4%* | *0* | *3* |
|  | *Ok* | *147* | *5.2%* | *0* | *3* |
|  | *Good* | *10* | *0.4%* | *0* | *3* |
|  | *Total* | *2830* | *100.0* |  |  |
| *Medical support if got COVID?* | *No* | *2578* | *91.1%* | *0* | *1* |
|  | *Yes* | *252* | *8.9%* | *0* | *1* |
|  | *Total* | *2830* | *100.0* |  |  |
| *Community support if got COVID?* | *No* | *2635* | *93.1%* | *0* | *1* |
|  | *Yes* | *195* | *6.9%* | *0* | *1* |
|  | *Total* | *2830* | *100.0* |  |  |
| *Government support if got COVID?* | *No* | *2604* | *92.0%* | *0* | *1* |
|  | *Yes* | *226* | *8.0%* | *0* | *1* |
|  | *Total* | *2830* | *100.0* |  |  |
| *Paid rent during lockdown?* | *No* | *1337* | *47.2%* | *0* | *1* |
|  | *Yes* | *1493* | *52.8%* | *0* | *1* |
|  | *Total* | *2830* | *100.0* |  |  |
| *Changed to poorer locality after lockdown?* | *No* | *2281* | *80.6%* | *0* | *1* |
|  | *Yes* | *549* | *19.4%* | *0* | *1* |
|  | *Total* | *2830* | *100.0* |  |  |
| *Moved to smaller house after lockdown?* | *No* | *2351* | *83.1%* | *0* | *1* |
|  | *Yes* | *479* | *16.9%* | *0* | *1* |
|  | *Total* | *2830* | *100.0* |  |  |
| *Where were you during lockdown?* | *No* | *552* | *19.5%* | *0* | *1* |
|  | *Yes* | *2278* | *80.5%* | *0* | *1* |
|  | *Total* | *2830* | *100.0* |  |  |
| *Did you get the old job back after lockdown?* | *No* | *1174* | *41.5%* | *0* | *1* |
|  | *Yes* | *1656* | *58.5%* | *0* | *1* |
|  | *Total* | *2830* | *100.0* |  |  |
| *Did you learn something new for your current job? Skill* | *No* | *1464* | *51.7%* | *0* | *1* |
|  | *Yes* | *1366* | *48.3%* | *0* | *1* |
|  | *Total* | *2830* | *100.0* |  |  |
| *Did you learn something new for your current job? Technique* | *No* | *2385* | *84.3%* | *0* | *1* |
|  | *Yes* | *445* | *15.7%* | *0* | *1* |
|  | *Total* | *2830* | *100.0* |  |  |
| *Did you learn something new for your current job? Technology* | *No* | *2221* | *78.5%* | *0* | *1* |
|  | *Yes* | *609* | *21.5%* | *0* | *1* |
|  | *Total* | *2830* | *100.0* |  |  |

*Table 7 shows the impact of COVID-19 on the household environment, focusing on basic amenities such as garbage disposal, electricity, bathroom access, water availability, and cooking gas during the lockdown, reflecting the challenges households faced in maintaining essential services and daily living conditions.*

*Table 7 Descriptive statistics of the impact of COVID-19 on environment*

| *PART A* ***–*** *Impact of Covid on Environment* | | ***Frequency*** | ***Percent*** | ***Minimum*** | ***Maximum*** |
| --- | --- | --- | --- | --- | --- |
| *Was the garbage taken care of during lockdown?* | *No* | *781* | *27.6%* | *0* | *1* |
|  | *Yes* | *2049* | *72.4%* | *0* | *1* |
|  | *Total* | *2830* | *100.0* |  |  |
| *Had power at home at all time from March 2020?* | *No* | *703* | *24.8%* | *0* | *1* |
|  | *Yes* | *2127* | *75.2%* | *0* | *1* |
|  | *Total* | *2830* | *100.0* |  |  |
| *Have access to bathroom.* | *No* | *875* | *30.9%* | *0* | *1* |
|  | *Yes* | *1955* | *69.1%* | *0* | *1* |
|  | *Total* | *2830* | *100.0* |  |  |
| *Have access to water in the bathrooms.* | *No* | *1044* | *36.9%* | *0* | *1* |
|  | *Yes* | *1786* | *63.1%* | *0* | *1* |
|  | *Total* | *2830* | *100.0* |  |  |
| *Easy access to drinking water as before?* | *No* | *832* | *29.4%* | *0* | *1* |
|  | *Yes* | *1998* | *70.6%* | *0* | *1* |
|  | *Total* | *2830* | *100.0* |  |  |
| *Cooking gas was available during lockdown?* | *No* | *994* | *35.1%* | *0* | *1* |
|  | *Yes* | *1836* | *64.9%* | *0* | *1* |
|  | *Total* | *2830* | *100.0* |  |  |

*Table 8 illustrates the impact of the pandemic on food and nutrition intake among households. The table captures experiences of hunger, affordability of essential foods, worries about food availability, and the frequency of consumption of staples such as rice, wheat, milk/curd, dal/sambar, vegetables, and non-vegetarian items before and during the pandemic. The table highlights changes in dietary patterns and food security challenges faced by families during lockdown and afterwards.*

*Table 8 Descriptive statistics of the* *impact of COVID-19 on food and nutrition intake*

| *PART A* ***–*** *Food and Nutrition* | | ***Frequency*** | ***Percent*** | ***Minimum*** | ***Maximum*** |
| --- | --- | --- | --- | --- | --- |
| *Did your family sleep hungry any time during the lockdown?* | *No* | *1945* | *68.7%* | *0* | *1* |
|  | *Yes* | *885* | *31.3%* | *0* | *1* |
|  | *Total* | *2830* | *100.0* |  |  |
| *Did your family sleep hungry any time after the lockdown?* | *No* | *2439* | *86.2%* | *0* | *1* |
|  | *Yes* | *391* | *13.8%* | *0* | *1* |
|  | *Total* | *2830* | *100.0* |  |  |
| *Any food which family needs but cannot afford right now?* | *No* | *1940* | *68.6%* | *0* | *1* |
|  | *Vegetables* | *213* | *7.5%* | *0* | *1* |
|  | *Fruits* | *29* | *1.0%* | *0* | *1* |
|  | *Dairy products* | *105* | *3.7%* | *0* | *1* |
|  | *Cereals (Rice)* | *65* | *2.3%* | *0* | *1* |
|  | *Pulses (lentils/Dals)* | *10* | *0.4%* | *0* | *1* |
|  | *Non-veg/Meats/fish* | *133* | *4.7%* | *0* | *1* |
|  | *Yes, Difficulty in more than 2 major food groups* | *335* | *11.8%* | *0* | *1* |
|  | *Total* | *2830* | *100.0* |  |  |
| *During lockdown did you ever worry that the family will not have enough food? If yes, why did you worry?* | *No* | *2034* | *71.9%* | *0* | *3* |
|  | *Financial problem because of no job/work* | *710* | *25.1%* | *0* | *3* |
|  | *Fear of getting corona* | *25* | *0.9%* | *0* | *3* |
|  | *Worry about future/children future* | *61* | *2.2%* | *0* | *3* |
|  | *Total* | *2830* | *100.0* |  |  |
| *Rice/Wheat-Daily- (2018-2019)* | *No* | *1217* | *43.0%* | *0* | *1* |
|  | *Yes* | *1613* | *57.0%* | *0* | *1* |
|  | *Total* | *2830* | *100.0* |  |  |
| *Rice/Wheat-1-3 times a week- (2018-1019)* | *No* | *2186* | *77.2%* | *0* | *1* |
|  | *Yes* | *644* | *22.8%* | *0* | *1* |
|  | *Total* | *2830* | *100.0* |  |  |
| *Rice/Wheat-weekly- (2018-2019)* | *No* | *2581* | *91.2%* | *0* | *1* |
|  | *Yes* | *249* | *8.8%* | *0* | *1* |
|  | *Total* | *2830* | *100.0* |  |  |
| *Milk/Curd-Daily- (2018-2019)* | *No* | *786* | *27.8%* | *0* | *1* |
|  | *Yes* | *2044* | *72.2%* | *0* | *1* |
|  | *Total* | *2830* | *100.0* |  |  |
| *Milk/Curd-1-3 times a week- (1018-1019)* | *No* | *2611* | *92.3%* | *0* | *1* |
|  | *Yes* | *219* | *7.7%* | *0* | *1* |
|  | *Total* | *2830* | *100.0* |  |  |
| *Milk/Curd-weekly- (2018-2019)* | *No* | *2697* | *95.3%* | *0* | *1* |
|  | *Yes* | *133* | *4.7%* | *0* | *1* |
|  | *Total* | *2830* | *100.0* |  |  |
| *Sambar/Dal-Daily- (2018-2019)* | *No* | *1294* | *45.7%* | *0* | *1* |
|  | *Yes* | *1536* | *54.3%* | *0* | *1* |
|  | *Total* | *2830* | *100.0* |  |  |
| *Sambar/Dal-1-3 times a week- (1018-1019)* | *No* | *2154* | *76.1%* | *0* | *1* |
|  | *Yes* | *676* | *23.9%* | *0* | *1* |
|  | *Total* | *2830* | *100.0* |  |  |
| *Sambar/Dal-weekly- (2018-2019)* | *No* | *2585* | *91.3%* | *0* | *1* |
|  | *Yes* | *245* | *8.7%* | *0* | *1* |
|  | *Total* | *2830* | *100.0* |  |  |
| *Nonveg-Daily- (2018-2019)* | *No* | *2573* | *90.9%* | *0* | *1* |
|  | *Yes* | *257* | *9.1%* | *0* | *1* |
|  | *Total* | *2830* | *100.0* |  |  |
| *Nonveg-1-3 times a week- (2018-1019)* | *No* | *2186* | *77.2%* | *0* | *1* |
|  | *Yes* | *644* | *22.8%* | *0* | *1* |
|  | *Total* | *2830* | *100.0* |  |  |
| *Nonveg-weekly- (2018-2019)* | *No* | *1413* | *49.9%* | *0* | *1* |
|  | *Yes* | *1417* | *50.1%* | *0* | *1* |
|  | *Total* | *2830* | *100.0* |  |  |
| *Vegetables-Daily- (2018-2019)* | *No* | *2143* | *75.7%* | *0* | *1* |
|  | *Yes* | *687* | *24.3%* | *0* | *1* |
|  | *Total* | *2830* | *100.0* |  |  |
| *Vegetables-1-3 times a week- (2018-1019)* | *No* | *1745* | *61.7%* | *0* | *1* |
|  | *Yes* | *1085* | *38.3%* | *0* | *1* |
|  | *Total* | *2830* | *100.0* |  |  |
| *Vegetables-weekly- (2018-2019)* | *No* | *2164* | *76.5%* | *0* | *1* |
|  | *Yes* | *666* | *23.5%* | *0* | *1* |
|  | *Total* | *2830* | *100.0* |  |  |
| *Rice and Wheat-Daily-(2020 to now)* | *No* | *1520* | *53.7%* | *0* | *1* |
|  | *Yes* | *1310* | *46.3%* | *0* | *1* |
|  | *Total* | *2830* | *100.0* |  |  |
| *Rice and Wheat-1-3 times a week-(2020 to now)* | *No* | *1830* | *64.7%* | *0* | *1* |
|  | *Yes* | *1000* | *35.3%* | *0* | *1* |
|  | *Total* | *2830* | *100.0* |  |  |
| *Rice and Wheat-Weekly-(2020 to now)* | *No* | *2545* | *89.9%* | *0* | *1* |
|  | *Yes* | *285* | *10.1%* | *0* | *1* |
|  | *Total* | *2830* | *100.0* |  |  |
| *Milk/Curd-Daily-(2020 to now)* | *No* | *1320* | *46.6%* | *0* | *1* |
|  | *Yes* | *1510* | *53.4%* | *0* | *1* |
|  | *Total* | *2830* | *100.0* |  |  |
| *Milk/Curd-1-3 times a week-(2020 to now)* | *No* | *2148* | *75.9%* | *0* | *1* |
|  | *Yes* | *682* | *24.1%* | *0* | *1* |
|  | *Total* | *2830* | *100.0* |  |  |
| *Milk/Curd-Weekly-(2020 to now)* | *No* | *2598* | *91.8%* | *0* | *1* |
|  | *Yes* | *232* | *8.2%* | *0* | *1* |
|  | *Total* | *2830* | *100.0* |  |  |
| *Sambar/Dal-Daily-(2020 to now)* | *No* | *1581* | *55.9%* | *0* | *1* |
|  | *Yes* | *1249* | *44.1%* | *0* | *1* |
|  | *Total* | *2830* | *100.0* |  |  |
| *Sambar/Dal-1-3 times a week-(2020 to now)* | *No* | *1845* | *65.2%* | *0* | *1* |
|  | *Yes* | *985* | *34.8%* | *0* | *1* |
|  | *Total* | *2830* | *100.0* |  |  |
| *Sambar/Dal-Weekly-(2020 to now)* | *No* | *2477* | *87.5%* | *0* | *1* |
|  | *Yes* | *353* | *12.5%* | *0* | *1* |
|  | *Total* | *2830* | *100.0* |  |  |
| *Nonveg-Daily-(2020 to now)* | *No* | *2687* | *94.9%* | *0* | *1* |
|  | *Yes* | *143* | *5.1%* | *0* | *1* |
|  | *Total* | *2830* | *100.0* |  |  |
| *Nonveg-1-3 times a week-(2020 to now)* | *No* | *2396* | *84.7%* | *0* | *1* |
|  | *Yes* | *434* | *15.3%* | *0* | *1* |
|  | *Total* | *2830* | *100.0* |  |  |
| *Nonveg-Weekly-(2020 to now)* | *No* | *1199* | *42.4%* | *0* | *1* |
|  | *Yes* | *1631* | *57.6%* | *0* | *1* |
|  | *Total* | *2830* | *100.0* |  |  |
| *Veg-Daily-(2020 to now)* | *No* | *2389* | *84.4%* | *0* | *1* |
|  | *Yes* | *441* | *15.6%* | *0* | *1* |
|  | *Total* | *2830* | *100.0* |  |  |
| *Veg-1-3 times a week-(2020 to now)* | *No* | *1765* | *62.4%* | *0* | *1* |
|  | *Yes* | *1065* | *37.6%* | *0* | *1* |
|  | *Total* | *2830* | *100.0* |  |  |
| *Veg-Weekly-(2020 to now)* | *No* | *1765* | *62.4%* | *0* | *1* |
|  | *Yes* | *1065* | *37.6%* | *0* | *1* |
|  | *Total* | *2830* | *100.0* |  |  |

*Table 9 shows the impact of the pandemic on personal and community environments. The table covers changes in work practices, technology use, and wages, as well as emotional and behavioural wellbeing during COVID-19. The table also reflects the type of support received from the government during and after the lockdown, along with people’s expectations of what more could have been done to support them.*

*Table 9 Descriptive statistics of the impact of COVID-19 on personal and community environments*

| *PART A* ***–*** *Personal and Community Environment* | | ***Frequency*** | ***Percent*** | ***Minimum*** | ***Maximum*** |
| --- | --- | --- | --- | --- | --- |
| *Are people using more machines/technology in your job, after lockdown?* | *No* | *1577* | *55.7%* | *0* | *1* |
|  | *Yes* | *1253* | *44.3%* | *0* | *1* |
|  | *Total* | *2830* | *100.0* |  |  |
| *What do you use your mobile for? -Watch news* | *No* | *1734* | *61.3%* | *0* | *1* |
|  | *Yes* | *1096* | *38.7%* | *0* | *1* |
|  | *Total* | *2830* | *100.0* |  |  |
| *What do you use your mobile for? -Watch TV programs* | *No* | *1521* | *53.7%* | *0* | *1* |
|  | *Yes* | *1309* | *46.3%* | *0* | *1* |
|  | *Total* | *2830* | *100.0* |  |  |
| *What do you use your mobile for? -YouTube and other videos* | *No* | *1540* | *54.4%* | *0* | *1* |
|  | *Yes* | *1290* | *45.6%* | *0* | *1* |
|  | *Total* | *2830* | *100.0* |  |  |
| *Has nature of your job changed since COVID? -More Technical* | *No* | *2256* | *79.7%* | *0* | *1* |
|  | *Yes* | *574* | *20.3%* | *0* | *1* |
|  | *Total* | *2830* | *100.0* |  |  |
| *Has nature of your job changed since COVID? -More use of phone* | *No* | *1838* | *64.9%* | *0* | *1* |
|  | *Yes* | *992* | *35.1%* | *0* | *1* |
|  | *Total* | *2830* | *100.0* |  |  |
| *Has nature of your job changed since COVID? -Less time to work/have to work faster* | *No* | *2405* | *85.0%* | *0* | *1* |
|  | *Yes* | *425* | *15.0%* | *0* | *1* |
|  | *Total* | *2830* | *100.0* |  |  |
| *Has nature of your job changed since COVID? -Less work* | *No* | *1672* | *59.1%* | *0* | *1* |
|  | *Yes* | *1158* | *40.9%* | *0* | *1* |
|  | *Total* | *2830* | *100.0* |  |  |
| *Has nature of your job changed since COVID? -Had to pay deposit* | *No* | *2576* | *91.0%* | *0* | *1* |
|  | *Yes* | *254* | *9.0%* | *0* | *1* |
|  | *Total* | *2830* | *100.0* |  |  |
| *Has nature of your job changed since COVID? -Wages are paid late* | *No* | *2304* | *81.4%* | *0* | *1* |
|  | *Yes* | *526* | *18.6%* | *0* | *1* |
|  | *Total* | *2830* | *100.0* |  |  |
| *Has nature of your job changed since COVID? -Weges have reduced* | *No* | *2186* | *77.2%* | *0* | *1* |
|  | *Yes* | *644* | *22.8%* | *0* | *1* |
|  | *Total* | *2830* | *100.0* |  |  |
| *Do you feel more angry or irritated these days?* | *No* | *499* | *17.6%* | *0* | *2* |
|  | *A little* | *2314* | *81.8%* | *0* | *2* |
|  | *Yes* | *17* | *0.6%* | *0* | *2* |
|  | *Total* | *2830* | *100.0* |  |  |
| *Do you feel like crying or feel low these days?* | *No* | *2184* | *77.2%* | *0* | *1* |
|  | *Yes* | *646* | *22.8%* | *0* | *1* |
|  | *Total* | *2830* | *100.0* |  |  |
| *Have been there times last year when you did not like to take bath or change clothes for a week/ weeks?* | *No* | *1644* | *58.1%* | *0* | *2* |
|  | *A little* | *958* | *33.9%* | *0* | *2* |
|  | *Yes* | *228* | *8.1%* | *0* | *2* |
|  | *Total* | *2830* | *100.0* |  |  |
| *Support from government during lockdown* | *No ration/no money/no helpline/no medical* | *974* | *34.4%* | *0* | *5* |
|  | *Ration* | *1186* | *41.9%* | *0* | *5* |
|  | *Medical help* | *233* | *8.2%* | *0* | *5* |
|  | *Money in the account* | *117* | *4.1%* | *0* | *5* |
|  | *Help line to call if needed* | *173* | *6.1%* | *0* | *5* |
|  | *Helpless/self-account* | *147* | *5.2%* | *0* | *5* |
|  | *Total* | *2830* | *100.0* |  |  |
| *Support from government after the lockdown (May-Aug 2020)?* | *No ration/no money/no helpline/no medical* | *927* | *32.8%* | *0* | *5* |
|  | *Ration* | *1368* | *48.3%* | *0* | *5* |
|  | *Medical help* | *271* | *9.6%* | *0* | *5* |
|  | *Money in the account* | *141* | *5.0%* | *0* | *5* |
|  | *Help line to call if needed* | *90* | *3.2%* | *0* | *5* |
|  | *Helpless/self-account* | *33* | *1.2%* | *0* | *5* |
|  | *Total* | *2830* | *100.0* |  |  |
| *What do you think government could have done more to support you?* | *Nothing/no expectation/not satisfy of government* | *1167* | *41.2%* | *0* | *4* |
|  | *Ration/food items* | *594* | *21.0%* | *0* | *4* |
|  | *Medical help/covid will be stop/health awareness* | *192* | *6.8%* | *0* | *4* |
|  | *Financial help and job help/loan/reduce school fees/reduce rent* | *825* | *29.2%* | *0* | *4* |
|  | *Needs like wastage management/ help to migrant/support senior people* | *52* | *1.8%* | *0* | *4* |
|  | *Total* | *2830* | *100.0* |  |  |

*Table 10 includes Part B of the questionnaire and summarizes gig workers’ fears and apprehensions during COVID-19. The table captures concerns about wages, job security, and loan repayment, as well as social and community dynamics such as neighbour and community support, local tensions, family violence, and increased drinking. The table also highlights technology-related anxieties, mobility patterns, participation in family events, postponed ceremonies, and landlord harassment experienced during the pandemic.*

*Table 10 Descriptive statistics of gig worker’s fears and apprehensions.*

| *PART B – Do You Think* | | ***Frequency*** | ***Percent*** | ***Minimum*** | ***Maximum*** |
| --- | --- | --- | --- | --- | --- |
| *Is there a possibility now of increase in wages from your employer?* | *No* | *1292* | *45.7%* | *1* | *5* |
|  | *Mostly No* | *230* | *8.1%* | *1* | *5* |
|  | *Not sure* | *324* | *11.4%* | *1* | *5* |
|  | *Mostly Yes* | *569* | *20.1%* | *1* | *5* |
|  | *Yes* | *415* | *14.7%* | *1* | *5* |
|  | *Total* | *2830* | *100.0* |  |  |
| *Will you be able to pay the loan back in case you borrow in an emergency?* | *No* | *195* | *6.9%* | *1* | *5* |
|  | *Mostly No* | *756* | *26.7%* | *1* | *5* |
|  | *Not sure* | *887* | *31.3%* | *1* | *5* |
|  | *Mostly Yes* | *522* | *18.4%* | *1* | *5* |
|  | *Yes* | *470* | *16.6%* | *1* | *5* |
|  | *Total* | *2830* | *100.0* |  |  |
| *Has COVID made you worry about job security, and fear of loss of job?* | *No* | *650* | *23.0%* | *1* | *5* |
|  | *Mostly No* | *183* | *6.5%* | *1* | *5* |
|  | *Not sure* | *382* | *13.5%* | *1* | *5* |
|  | *Mostly Yes* | *571* | *20.2%* | *1* | *5* |
|  | *Yes* | *1044* | *36.9%* | *1* | *5* |
|  | *Total* | *2830* | *100.0* |  |  |
| *Did you get any support from your neighbours during the lockdown and high COVID months? Emotional* | *No* | *2361* | *83.4%* | *0* | *1* |
|  | *Yes* | *469* | *16.6%* | *0* | *1* |
|  | *Total* | *2830* | *100.0* |  |  |
| *Did you get any support from your neighbours during the lockdown and high COVID months? -Information* | *No* | *1933* | *68.3%* | *0* | *1* |
|  | *Yes* | *897* | *31.7%* | *0* | *1* |
|  | *Total* | *2830* | *100.0* |  |  |
| *Did you get any support from your neighbours during the lockdown and high COVID months? -Money* | *No* | *2590* | *91.5%* | *0* | *1* |
|  | *Yes* | *240* | *8.5%* | *0* | *1* |
|  | *Total* | *2830* | *100.0* |  |  |
| *Did you get any support from your neighbours during the lockdown and high COVID months? -Food* | *No* | *1855* | *65.5%* | *0* | *1* |
|  | *Yes* | *975* | *34.5%* | *0* | *1* |
|  | *Total* | *2830* | *100.0* |  |  |
| *Did you get any support from your neighbours during the lockdown and high COVID months? -Care during illness* | *No* | *2709* | *95.7%* | *0* | *1* |
|  | *Yes* | *121* | *4.3%* | *0* | *1* |
|  | *Total* | *2830* | *100.0* |  |  |
| *Did you get any support from your community during the lockdown and high COVID months? -Emotional* | *No* | *2594* | *91.7%* | *0* | *1* |
|  | *Yes* | *236* | *8.3%* | *0* | *1* |
|  | *Total* | *2830* | *100.0* |  |  |
| *Did you get any support from your community during the lockdown and high COVID months? -Information* | *No* | *1967* | *69.5%* | *0* | *1* |
|  | *Yes* | *863* | *30.5%* | *0* | *1* |
|  | *Total* | *2830* | *100.0* |  |  |
| *Did you get any support from your community during the lockdown and high COVID months? -Money* | *No* | *2679* | *94.7%* | *0* | *1* |
|  | *Yes* | *151* | *5.3%* | *0* | *1* |
|  | *Total* | *2830* | *100.0* |  |  |
| *Did you get any support from your community during the lockdown and high COVID months? -Food* | *No* | *1619* | *57.2%* | *0* | *1* |
|  | *Yes* | *1211* | *42.8%* | *0* | *1* |
|  | *Total* | *2830* | *100.0* |  |  |
| *Did you get any support from your community during the lockdown and high COVID months? -Care during illness* | *No* | *2735* | *96.6%* | *0* | *1* |
|  | *Yes* | *95* | *3.4%* | *0* | *1* |
|  | *Total* | *2830* | *100.0* |  |  |
| *Did see any local versus outsider community tensions in the neighbourhood?* | *No* | *980* | *34.6%* | *1* | *5* |
|  | *Mostly No* | *427* | *15.1%* | *1* | *5* |
|  | *Not sure* | *737* | *26.0%* | *1* | *5* |
|  | *Mostly Yes* | *319* | *11.3%* | *1* | *5* |
|  | *Yes* | *367* | *13.0%* | *1* | *5* |
|  | *Total* | *2830* | *100.0* |  |  |
| *Did you see family violence increase in your locality during the lockdown months?* | *No* | *1058* | *37.4%* | *1* | *5* |
|  | *Mostly No* | *634* | *22.4%* | *1* | *5* |
|  | *Not sure* | *436* | *15.4%* | *1* | *5* |
|  | *Mostly Yes* | *307* | *10.8%* | *1* | *5* |
|  | *Yes* | *395* | *14.0%* | *1* | *5* |
|  | *Total* | *2830* | *100.0* |  |  |
| *Did you see increase in Drinking in the neighbourhood after the lockdown?* | *No* | *1292* | *45.7%* | *1* | *5* |
|  | *Mostly No* | *374* | *13.2%* | *1* | *5* |
|  | *Not sure* | *377* | *13.3%* | *1* | *5* |
|  | *Mostly Yes* | *396* | *14.0%* | *1* | *5* |
|  | *Yes* | *391* | *13.8%* | *1* | *5* |
|  | *Total* | *2830* | *100.0* |  |  |
| *Do you feel anxiety about use of more technology in your job?* | *No* | *1541* | *54.5%* | *1* | *5* |
|  | *Mostly No* | *266* | *9.4%* | *1* | *5* |
|  | *Not sure* | *353* | *12.5%* | *1* | *5* |
|  | *Mostly Yes* | *253* | *8.9%* | *1* | *5* |
|  | *Yes* | *417* | *14.7%* | *1* | *5* |
|  | *Total* | *2830* | *100.0* |  |  |
| *Did you visit your native place during high COVID months?* | *No* | *1817* | *64.2%* | *1* | *5* |
|  | *Mostly No* | *224* | *7.9%* | *1* | *5* |
|  | *Not sure* | *267* | *9.4%* | *1* | *5* |
|  | *Mostly Yes* | *222* | *7.8%* | *1* | *5* |
|  | *Yes* | *300* | *10.6%* | *1* | *5* |
|  | *Total* | *2830* | *100.0* |  |  |
| *Did you attend any family / social function during high COVID months?* | *No* | *1888* | *66.7%* | *1* | *5* |
|  | *Mostly No* | *203* | *7.2%* | *1* | *5* |
|  | *Not sure* | *348* | *12.3%* | *1* | *5* |
|  | *Mostly Yes* | *131* | *4.6%* | *1* | *5* |
|  | *Yes* | *260* | *9.2%* | *1* | *5* |
|  | *Total* | *2830* | *100.0* |  |  |
| *Did your family postponed marriage ceremony or any family function due to COVID?* | *No* | *1738* | *61.4%* | *1* | *5* |
|  | *Mostly No* | *168* | *5.9%* | *1* | *5* |
|  | *Not sure* | *200* | *7.1%* | *1* | *5* |
|  | *Mostly Yes* | *213* | *7.5%* | *1* | *5* |
|  | *Yes* | *511* | *18.1%* | *1* | *5* |
|  | *Total* | *2830* | *100.0* |  |  |
| *Do you face increased harassment from landlords after the lockdown?* | *No* | *1969* | *69.6%* | *1* | *5* |
|  | *Mostly No* | *114* | *4.0%* | *1* | *5* |
|  | *Not sure* | *260* | *9.2%* | *1* | *5* |
|  | *Mostly Yes* | *109* | *3.9%* | *1* | *5* |
|  | *Yes* | *378* | *13.4%* | *1* | *5* |
|  | *Total* | *2830* | *100.0* |  |  |

*Next, Table 11 presents responses on vaccine awareness and attitudes during COVID-19. The table covers knowledge about the vaccine, beliefs regarding its effectiveness, awareness of vaccination sites, and willingness to get vaccinated.*

*Table 11 Descriptive statistics on vaccine awareness and attitudes*

| *PART B – Vaccine* | | | ***Frequency*** | | ***Percent*** | | ***Minimum*** | | ***Maximum*** | |
| --- | --- | --- | --- | --- | --- | --- | --- | --- | --- | --- |
| *Are you aware that vaccine / injection is being given to stop COVID?* | *No* | | *873* | | *30.8%* | | *0* | | *1* |  |
|  | *Yes* | | *1957* | | *69.2%* | | *0* | | *1* |  |
|  | *Total* | | *2830* | | *100.0* | |  | |  |  |
| *Do you believe that it can cure COVID and save us from Corona?* | *No* | | *1139* | | *40.2%* | | *0* | | *2* |  |
|  | *Don’t know* | | *485* | | *17.1%* | | *0* | | *2* |  |
|  | *Yes* | | *1206* | | *42.6%* | | *0* | | *2* |  |
|  | *Total* | | *2830* | | *100.0* | |  | |  |  |
| *Do you know where the vaccine / injection is being given?* | *No* | | *1843* | | *65.1%* | | *0* | | *1* |  |
|  | *Yes* | | *987* | | *34.9%* | | *0* | | *1* |  |
|  | *Total* | | *2830* | | *100.0* | |  | |  |  |
| *Do you want to take the vaccine?* | *No* | | *1976* | | *69.8%* | | *0* | | *1* |  |
|  | *Yes* | | *854* | | *30.2%* | | *0* | | *1* |  |
|  | *Total* | | *2830* | | *100.0* | |  | |  |  |

*Table 12 presents the cross-tabulation of sociodemographic characteristics of low-income gig workers with their fears, apprehensions, and livelihood conditions across two phases: July–November 2019 and December 2020–January 2021.The variables include access to government support, job type, and household food intake patterns (rice/wheat, milk/curd, sambar/dal, non-vegetarian items, and vegetables). The results highlight a reduction in food consumption and changes in occupational distribution during the pandemic, alongside a shift in the type of government support received.*

*Table 12* *Crosstab results between variables before and during the pandemic*

| ***Variables of Interest*** | | ***Before COVID-19*** | | ***During COVID-19*** | |
| --- | --- | --- | --- | --- | --- |
|  |  | ***Count*** | ***Column N %*** | ***Count*** | ***Column N %*** |
| *Support from government* | *No ration/no money/no helpline/no medical* | *927* | *32.8%* | *974* | *34.4%* |
|  | *Ration* | *1368* | *48.3%* | *1186* | *41.9%* |
|  | *Medical help* | *271* | *9.6%* | *233* | *8.2%* |
|  | *Money in the account* | *141* | *5.0%* | *117* | *4.1%* |
|  | *Help line to call if needed* | *90* | *3.2%* | *173* | *6.1%* |
|  | *Helpless/self-account* | *33* | *1.2%* | *147* | *5.2%* |
| *Job* | *Not work/nothing* | *489* | *17.3%* | *37* | *1.3%* |
|  | *Drivers* | *486* | *17.2%* | *511* | *18.1%* |
|  | *Delivery* | *459* | *16.2%* | *501* | *17.7%* |
|  | *Beauty and other services* | *45* | *1.6%* | *111* | *3.9%* |
|  | *Street vendors* | *378* | *13.4%* | *719* | *25.4%* |
|  | *Domestic workers* | *725* | *25.6%* | *710* | *25.1%* |
|  | *Small Business* | *168* | *5.9%* | *169* | *6.0%* |
|  | *Self-employed* | *80* | *2.8%* | *72* | *2.5%* |
| *Food Intake- Rice/Wheat-Daily* | *No* | *1217* | *43.0%* | *1520* | *53.7%* |
|  | *Yes* | *1613* | *57.0%* | *1310* | *46.3%* |
| *Food Intake- Rice/Wheat-1-3 times a week* | *No* | *2186* | *77.2%* | *1830* | *64.7%* |
|  | *Yes* | *644* | *22.8%* | *1000* | *35.3%* |
| *Food Intake- Rice/Wheat-weekly* | *No* | *2581* | *91.2%* | *2545* | *89.9%* |
|  | *Yes* | *249* | *8.8%* | *285* | *10.1%* |
| *Food Intake- Milk/Curd-Daily* | *No* | *786* | *27.8%* | *1320* | *46.6%* |
|  | *Yes* | *2044* | *72.2%* | *1510* | *53.4%* |
| *Food Intake- Milk/Curd-1-3 times a week* | *No* | *2611* | *92.3%* | *2148* | *75.9%* |
|  | *Yes* | *219* | *7.7%* | *682* | *24.1%* |
| *Food Intake- Milk/Curd-weekly* | *No* | *2697* | *95.3%* | *2598* | *91.8%* |
|  | *Yes* | *133* | *4.7%* | *232* | *8.2%* |
| *Food Intake- Sambar/Dal-Daily* | *No* | *1294* | *45.7%* | *1581* | *55.9%* |
|  | *Yes* | *1536* | *54.3%* | *1249* | *44.1%* |
| *Food Intake- Sambar/Dal-1-3 times a week* | *No* | *2154* | *76.1%* | *1845* | *65.2%* |
|  | *Yes* | *676* | *23.9%* | *985* | *34.8%* |
| *Food Intake- Sambar/Dal-weekly* | *No* | *2585* | *91.3%* | *2477* | *87.5%* |
|  | *Yes* | *245* | *8.7%* | *353* | *12.5%* |
| *Food Intake- Nonveg-Daily* | *No* | *2573* | *90.9%* | *2687* | *94.9%* |
|  | *Yes* | *257* | *9.1%* | *143* | *5.1%* |
| *Food Intake- Nonveg-1-3 times a week* | *No* | *2186* | *77.2%* | *2396* | *84.7%* |
|  | *Yes* | *644* | *22.8%* | *434* | *15.3%* |
| *Food Intake- Nonveg-weekly* | *No* | *1413* | *49.9%* | *1199* | *42.4%* |
|  | *Yes* | *1417* | *50.1%* | *1631* | *57.6%* |
| *Food Intake- Vegetables-Daily* | *No* | *2143* | *75.7%* | *2389* | *84.4%* |
|  | *Yes* | *687* | *24.3%* | *441* | *15.6%* |
| *Food Intake- Vegetables-1-3 times a week* | *No* | *1745* | *61.7%* | *1765* | *62.4%* |
|  | *Yes* | *1085* | *38.3%* | *1065* | *37.6%* |
| *Food Intake- Vegetables-weekly* | *No* | *2164* | *76.5%* | *1765* | *62.4%* |
|  | *Yes* | *666* | *23.5%* | *1065* | *37.6%* |
